# Adenomyosis and Adolescence: A Challenging Diagnosis and Complex Management

**DOI:** 10.3390/diagnostics14212344

**Published:** 2024-10-22

**Authors:** Francesco Giuseppe Martire, Claudia d’Abate, Giorgia Schettini, Giulia Cimino, Alessandro Ginetti, Irene Colombi, Alberto Cannoni, Gabriele Centini, Errico Zupi, Lucia Lazzeri

**Affiliations:** Department of Molecular and Developmental Medicine, Obstetrics and Gynecological Clinic, University of Siena, Strada delle Scotte 14, 53100 Siena, Italy; francescogmartire@libero.it (F.G.M.); claudiadabate94@gmail.com (C.d.); giorgiaschettini@gmail.com (G.S.); giulia.cimino@student.unisi.it (G.C.); ginettialessandro14@gmail.com (A.G.); colombi.irene1@gmail.com (I.C.); albertoacannoni@gmail.com (A.C.); centini.gabriele@gmail.com (G.C.); lucialazzeri79@gmail.com (L.L.)

**Keywords:** adolescence, adenomyosis, diagnosis, management, symptoms

## Abstract

Adenomyosis is a chronic, hormone-related disease characterized by the presence of the endometrial glands and stroma within the myometrium. This condition can manifest in various features, focal or diffuse adenomyosis or as an adenomyoma, and it may involve different uterine walls (posterior, anterior, and/or lateral walls). The disease can also be classified into different degrees, as mild, moderate and severe, which can be associated with more intense symptoms, although this correlation is not always directly proportional. In fact, adenomyosis can be asymptomatic in about a third of cases or it can significantly impact patients’ quality of life through painful symptoms, such as dysmenorrhea and dyspareunia, abnormal uterine bleeding—particularly heavy menstrual bleeding—and potential effects on fertility. Historically, adenomyosis has been considered a disease primarily affecting premenopausal women over the age of 40, often multiparous, because the diagnosis was traditionally based on surgical reports from hysterectomies performed after the completion of reproductive desire. Data on the presence of adenomyosis in adolescent patients remain limited. However, in recent years, advancements in noninvasive diagnostic tools and increased awareness of this pathology have enabled earlier diagnoses. The disease appears to have an early onset during adolescence, with a tendency to progress in terms of extent and severity over time. Adenomyosis often coexists with endometriosis, which also has an early onset. Therefore, it is important, when diagnosing adenomyosis, to also screen for concomitant endometriosis, especially deep endometriosis in the posterior compartment. The aim of this narrative review is to investigate the prevalence of different types and degrees of adenomyosis in younger patients, assess the associated symptoms, and describe the most appropriate diagnostic procedures for effective therapeutic management and follow-up, with the goal of improving the quality of life for these young women.

## 1. Introduction

Adenomyosis is a chronic benign disease characterized by the presence of endometrial tissue and glandular components within the myometrium. First described by von Rokintansky in 1860 as “some fibrous tumors of the uterus contain gland-like structures that resemble endometrial glands”, its definition has remained largely unchanged over time. The clinical symptoms, first detailed by Cullen in the early 20th century, have also remained consistent, including heavy menstrual bleeding (HMB) and various form of pelvic pain, such as dysmenorrhea, dyspareunia, and chronic pelvic pain [1,2,3].

The reported prevalence of adenomyosis varies widely, ranging from 1% to 70% across different studies, primarily because most epidemiological data have been based on surgical reports. Historically, adenomyosis has been considered a typical condition of multiparous women over more 40 years who underwent hysterectomy due to painful symptoms and/or HMB after completing their reproductive desires [4,5,6,7].

Until recently, despite significant interest in this disease, there were limited data regarding adenomyosis in adolescence. However, with the advancement of noninvasive diagnostic techniques, such as ultrasound (US) and magnetic resonance imaging (MRI), and the increasing need for early diagnosis to ensure appropriate management and improve quality of life, more data on adenomyosis in younger patients have emerged in recent years [5,8]. These new findings suggest that adenomyosis has an early onset, reflecting a range of pathogenetic factors beyond just uterine trauma and hyperperistalsis. Additionally, concomitant endometriosis is present in approximately 50% of cases, often with overlapping symptoms [5,9,10,11].

Given the disabling nature of this early-onset pathology, it is crucial to promote early non-invasive diagnosis and appropriate therapeutic management to improve quality of life, reduce disease progression, and preserve future reproductive potential [12].

The aim of this narrative review is to investigate the prevalence of different types and degrees of adenomyosis in young patients, evaluate the associated symptoms and explore potential differences in diagnostic and therapeutic management compared to pre-menopausal women.

## 2. Materials and Methods

**Figure 1:**
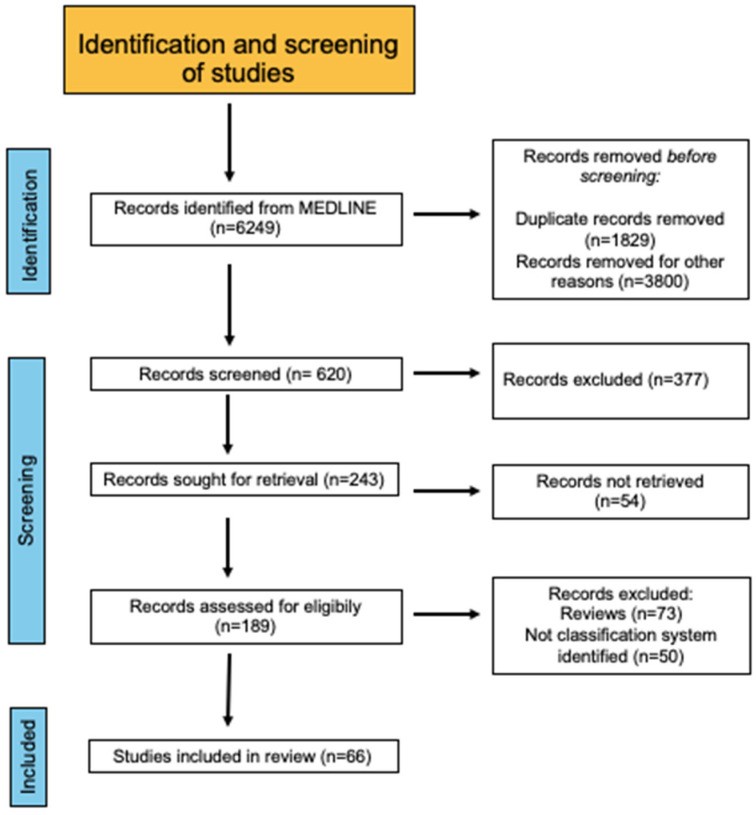
Flow diagram of study identification and selection.

An electronic literature search using the MEDLINE database was performed, aimed at identifying all English-language papers focusing on adenomyosis in adolescence from inception to April 2024 (Figure 1). Advanced literature search techniques were employed using “AND” and “OR” operators to refine or broaden the search scope. A combination of Medical Subject Headings (MeSH) search terms and of the following keywords were used to screen studies:

- (“Adenomyosis” OR “adenomyoma”) AND (“adolescence” OR “adolescent population” OR “adolescents” OR “young women”);
- (“Endometriosis” OR “endometriomas” OR “OMA”) AND (“adolescence” OR “adolescent population” OR “adolescents” OR “young women”);
- (“Adenomyosis” OR “adenomyoma”) AND (“adolescence” OR “adolescent population” OR “adolescents” OR “young women”) AND (“Diagnosis” OR “ultrasound” OR “ultrasonography”);
- (“Adenomyosis” OR “adenomyoma”) AND (“adolescence” OR “adolescent population” OR “adolescents” OR “young women”) AND (“Diagnosis” OR “magnetic resonance imaging” OR “ultrasonography”);
- (“Adenomyosis” OR “adenomyoma”) AND (“adolescence” OR “adolescent population” OR “adolescents” OR “young women”) AND (“Management” OR “Therapy”).

In this review, clinical trials (randomized and non-randomized), retrospective cohort studies, case-control studies, and observational prospective studies were considered for inclusion. The articles identified with the above-mentioned research were deemed eligible if they met the scope of this narrative review, i.e., to outline the clinical aspects of adenomyosis in adolescence and young adulthood (patients aged 12–25 years). Two authors (F.G.M. and L.L.) conducted the research independently and subsequently analyzed all the articles that met the inclusion criteria. The articles focusing on adenomyosis in adult populations (patients aged > 25 years) were excluded from this study. Ultimately, we included 106 articles in this review, published from 1995 to 2024.

All the features of the research topic were analyzed, starting with pathogenetic theories, followed by the epidemiological aspects of the disease, imaging techniques for the diagnosis, and concluding with the potential treatment options.

## 3. Results

### 3.1. Pathogenesis of Adenomyosis

There are several pathogenetic theories regarding adenomyosis. Among the most significant hypotheses there are the presence of genetic and epigenetic mutations, the theory of injury and tissue repair (TIAR), and the theory of development from metaplasia [11,13,14,15]. Genetic mutations, such as those in the *KRAS* and *PIK3CA* genes, are essential for enabling the uncontrolled proliferation of the epithelial component. However, this proliferative ability alone insufficient; a suitable microenvironment is also necessary to support this growth. In this context, the epigenetic mutations found in patients with adenomyosis are pivotal. Mechanisms like methylation, demethylation, and histone modification can lead to the overexpression or underexpression of specific proteins, creating a local environment of hyperestrogenism and reduced progestin sensitivity. Once an adenomyotic lesion forms, it undergoes cyclical bleeding similar to that of the eutopic endometrium, effectively becoming a site of repeated tissue repair (ReTIAR) [16,17]. This process progresses to fibrosis through epithelial–mesenchymal transition (EMT), fibroblast-to-myofibroblast transdifferentiation (FMT), and smooth muscle metaplasia (SMM) [18,19]. Recently, a hypothesis has emerged suggesting that Schwann cells (SCs) may play a role in the development of adenomyotic lesions. The interface zone between the myometrium and endometrium (EMI) harbors endometrial stem cells [20,21] and is richly innervated by peripheral nerves lined with Schwann cells (SCs) [22]. Indeed, an adenomyotic lesion could possibly originate from dedifferentiated Schwann cells (dSCs). Following injury, SCs dedifferentiate into an unmyelinated state [23], leading to changes in the EMI conveyed by the overexpression of various genes correlated with tissue repair [24]. These changes are collectively known as myometrial interface disruption (EMID). A hypothesis on the pivotal role of dSCs in this process has been formulated, stating that dedifferentiated Schwann cells might differentiate into endometrial epithelial cells following multiple stimuli by growth factors, estrogen, and inflammatory cytokines [25,26].

### 3.2. Prevalence in Adolescence

In the past, adenomyosis was mainly diagnosed post-operatively in adult women through histological evaluation, often after hysterectomy for severe symptoms. This reinforced the belief that it was a late-onset disease, with limited data on its prevalence in adolescents due to the unsuitability of this diagnostic method for younger patients. However, in the last decade, improvements in non-invasive diagnostic methods, such as ultrasound and magnetic resonance imaging (MRI), have prompted a reevaluation of the age of onset for this disease [27]. Several researchers have focused on identifying ultrasound and MRI criteria that enable a non-invasive diagnosis of adenomyosis. The literature suggests that adenomyosis may have a significant prevalence in younger populations, though the data are heterogeneous and vary depending on risk factors. For instance, when considering factors like painful symptoms (dysmenorrhea, dyspareunia, and chronic pelvic pain) and heavy menstrual bleeding (HMB), prevalence estimates range from 5.2% to 50%. In 2015, Pinzauti et al. [28] evaluated 205 women aged 18–30 years and reported a 34% prevalence of diffuse adenomyosis. A closer look at the sample reveals that all participants attended a contraception outpatient clinic, and 83% reported at least one painful symptom, which likely explains the high prevalence rate. In 2020, Martire et al. [27] assessed the prevalence of ultrasound signs of endometriosis and adenomyosis in adolescents aged 12–20 years. Of the 270 patients who underwent endocavitary ultrasound evaluation (either transvaginal or transrectal for those who were not sexually active), 5.2% displayed ultrasound signs of adenomyosis. The study also found that adolescents reporting dysmenorrhea or HMB had higher prevalence rates of adenomyosis—8.8% and 13.6%, respectively. Given the significant role of dysmenorrhea, Martire et al. [8] conducted a 2023 study aimed at evaluating the prevalence of endometriosis and adenomyosis in adolescents and young women aged 12–25 years who reported severe dysmenorrhea (VAS > 7), thereby reducing the bias of primary dysmenorrhea, which is often mild to moderate. Among the 271 participants, 18.1% showed ultrasound signs of adenomyosis, with prevalence rising to 25.6% in those who also reported HMB. Both studies noted a trend of increasing prevalence with age, indicating disease progression.

In a sample of 95 patients, Vannuccini et al. [29] evaluated the prevalence of adenomyosis in adolescents and young women aged 13–25 years, finding a 27.4% prevalence in those with dysmenorrhea and HMB. Prevalence rates of adenomyosis using MRI as a diagnostic tool appear to closely match those obtained via ultrasound. For instance, in 2023, Chapron et al. [30] conducted a study on adolescents aged 12–20 with severe dysmenorrhea, finding a 17.4% prevalence of adenomyosis. The study also noted a trend of increasing prevalence with age. These epidemiological findings, derived from both ultrasound and MRI performed by expert operators, suggest a consistent prevalence pattern. Moreover, the presence of certain symptoms may serve as important indicators, warranting closer investigation.

### 3.3. Symptoms

Adenomyosis can significantly impact the quality of life of adolescents and young women, presenting with symptoms such as painful menstruation, heavy menstrual bleeding, and potential fertility issues. However, in about a third of cases, the condition may be asymptomatic. Recognizing the symptoms that could suggest the presence of adenomyosis is crucial [31]. Dysmenorrhea is a key symptom of adenomyosis and can be classified into primary and secondary types. Primary dysmenorrhea is functional pain without an organic cause, while secondary dysmenorrhea indicates an underlying condition, often related to endometriosis or adenomyosis. Primary dysmenorrhea is typically mild to moderate (VAS < 7), whereas secondary dysmenorrhea, particularly in the context of adenomyosis or endometriosis, is usually more severe (VAS > 7). In cases of severe dysmenorrhea, adenomyosis prevalence can reach up to 35% [3]. Dyspareunia is another significant symptom, though it is less prevalent in this population since many adolescents and young women are not sexually active. When dyspareunia is present, it often indicates the co-occurrence of deep retrocervical endometriosis. Symptoms like dysuria and dyschezia are less commonly associated with adenomyosis, but should not be overlooked, as they could signal endometriosis in the posterior (bowel involvement) or anterior (bladder involvement) compartments. Heavy menstrual bleeding (HMB) is another critical early-onset symptom and is as significant as severe dysmenorrhea. In adolescents with abnormal uterine bleeding (AUB), adenomyosis is a primary concern, with other organic causes being relatively rare. The prevalence of adenomyosis is about 13.3% in adolescents with HMB and increases to 27.4% in young women. A major issue is that key symptoms of adenomyosis—severe dysmenorrhea and HMB—are often underestimated, leading to delays in diagnosis and referral to specialized centers [2]. At this stage of life, adenomyosis is often focal and mild, requiring diagnosis by expert operators using ultrasound or MRI.

### 3.4. Type and Degree

In our recent study [27], adenomyosis was identified in 5.2% of adolescent cases, with this percentage rising to 20% in the presence of risk factors. An ultrasound analysis of adenomyosis type and location in adults revealed a correlation between symptoms and severity. This finding supports earlier studies indicating that the severity of symptoms and clinical characteristics are related to the extent and depth of adenomyosis [5,32,33,34,35]. Using transvaginal ultrasound (TVUS), adenomyosis can be categorized into three types based on the extent of the disease within the uterus: diffuse adenomyosis, focal adenomyosis, and adenomyoma, which presents as a nodular lesion [5,36]. Each type can vary in the depth of involvement within the myometrial layers, affecting either the external or internal myometrium. Additionally, adenomyosis can be classified by severity into mild, moderate, or severe forms, reflecting the extent of the disease. These classifications are based on ultrasound signs such as the size of the adenomyotic foci, the thickness of the affected uterine walls, and the number of both direct and indirect ultrasound signs of disease. In some cases, features of adenomyosis are linked to symptom severity. For instance, severe dysmenorrhea and heavy menstrual bleeding (HMB) are often associated with severe diffuse adenomyosis. Ultrasound characteristics of diffuse adenomyosis are more common in older women with HMB compared to those with focal disease. However, the severity of adenomyosis does not always match symptom intensity; patients with severe disease may have less intense symptoms than those with a milder form. This discrepancy may be partly due to coexisting conditions such as endometriosis [5]. The location of the disease also affects symptom type: external diffuse adenomyosis is associated with HMB, while internal adenomyosis is linked to dysmenorrhea. Regarding the impact of the disease on fertility and pregnancy, focal involvement of the internal myometrium seems to be more closely correlated with infertility and recurrent miscarriage. In contrast, involvement of the external myometrium and the junctional zone (JZ) appears to be more associated with potential pregnancy complications. Therefore, it is crucial to describe not only the presence of adenomyosis, but also the specific type (diffuse, focal, or adenomyoma), the involvement of the internal or external myometrium, the affected uterine wall (anterior, posterior, or lateral), and the degree of the disease (mild, moderate, or severe).

### 3.5. Noninvasive Diagnosis

In the past, the diagnosis of this pathology relied solely on histopathological examinations. Nowadays, imaging is essential for an accurate diagnosis and tailored management, which may include medical treatment [37] or conservative surgical options [38]. Currently, the majority of women with adenomyosis are managed medically, even without histological confirmation of the disease. The diagnosis of adenomyosis should begin with clinical suspicion, prompted by relevant symptoms and signs, as well as their impact on the patient’s quality of life, which often leads them to seek medical attention. Imaging is now the primary method for diagnosing adenomyosis. Although MRI was historically preferred, recent studies show that transvaginal ultrasound offers comparable diagnostic performance, with reported sensitivity and specificity rates of 89% and 86%, respectively [39]. Due to its widespread availability, ultrasound has become the preferred initial diagnostic tool, while MRI is reserved for cases that remain unclear after ultrasound evaluation [40].

#### 3.5.1. Ultrasound (US)

Transvaginal or transrectal ultrasound has become the first-line investigation technique for gynecological patients, as it is a low-cost, easily accessible method that also allows a dynamic examination [41] Transabdominal ultrasound is preferred in cases of large uteri or when a transvaginal approach is not possible [42].

In 2015, a consensus on the morphological uterus sonographic assessment (MUSA) was published to standardize the description of myometrial ultrasound images. The 2022 update categorized ultrasound signs of adenomyosis into direct and indirect signs [43,44,45]. Direct features indicate the presence of ectopic endometrial tissue in the myometrium, while indirect features are secondary to the presence of this tissue in the myometrium, such as muscular hypertrophy (globular uterus) or artefacts (e.g., shadowing) [46,47].

There is no consensus on the number of ultrasound features needed to diagnose adenomyosis [43].

The direct features of adenomyosis are:

- Myometrial cysts: oval cystic areas of any size, located in the myometrium, with anechogenic, low-level, or “ground glass” content;
- Hyperechogenic islands: hyperechoic areas of any size located in the myometrium, without contact with the endometrium;
- Echogenic subendometrial lines and buds: endometrial tissue of any shape extending or protruding into the myometrium.

The indirect features of adenomyosis are:

- Globular uterus: the uterus is approximately spherical, with its measured diameters (length/width/depth) being roughly equal;
- Asymmetrical myometrial thickening: A significant difference in thickness of at least 5 mm between the anterior and posterior myometrial walls;
- Fan-shaped shadowing: presence of hypoechogenic linear stripes, classified subjectively as mild, moderate, or strong;
- Translesional vascularity: blood vessels oriented perpendicularly to the endometrial cavity or crossing the lesion;
- Irregular junctional zone (JZ): Irregularity related to the presence of cysts, buds, or hyperechogenic areas;
- Interrupted JZ: a proportion of the JZ is not visible (<50% or ≥50%, based on the subjective assessment of the sonographer).

However, the MUSA consensus does not specify a sufficient number of direct and indirect criteria to definitively diagnose adenomyosis via ultrasound. Moreover, the relationship between the extent of adenomyotic lesions and the severity of symptoms requires further investigation.

#### 3.5.2. Magnetic Resonance Imaging (MRI)

Magnetic resonance imaging should be considered a second-level diagnostic examination for the diagnosis, providing accurate results while remaining non-invasive for the patient [48]. MRI has high sensitivity and specificity; sensitivity ranges from 88 to 93%, while specificity ranges from 67 to 91% [49,50,51,52,53,54].

Many authors have proposed the diagnosis of adenomyosis via MRI by assessing the JZ on T2-weighted sequences [55]: thickening of the JZ, described by a ratio of junctional zone thickness to myometrial thickness larger than 40% and by subtracting the maximum and minimum JZ thickness seen with MRI (JZmax-JZmin), resulting more than 5 mm. A JZ thicker than 12 mm often correlates with adenomyosis [51,52,53,54,55,56], whereas a thickness below 8 mm generally rules out this pathology [57].

However, the thickness of the JZ can be influenced by various factors (e.g., medication, phase of the menstrual cycle, reproductive status, and age), leading some authors to criticize the first two proposed diagnostic criteria [46,54,58].

In contrast, the JZmax-JZmin criteria above 5 mm appears less influenced by confounding factors [52,53,54,55,56,57,58,59].

Although the diagnosis of adenomyosis primarily relies on JZ features in MRI, both direct and indirect signs within the myometrium can also be assessed [52,53,54,55,56,57,58,59,60]. Adenomyosis typically appears as an area with low signal intensity on T2-weighted images, reflecting smooth muscle hyperplasia and heterotopic endometrial tissue, and may also present intramyometrial cysts. T1-weighted sequences are useful for identifying hemorrhage, with a high positive predictive value (95%) but low sensitivity (47.5%) [56].

A few years ago, a classification of adenomyosis based on MRI features was proposed [43,61], distinguishing internal adenomyosis, external adenomyosis, and adenomyoma. Internal adenomyosis can be classified as diffuse, superficial, or focal, while external adenomyosis is categorized as anterior or posterior. Following the development of the technique, new proposals for adenomyosis classification based on MRI findings were made [62], always focusing on the location of the disease in the myometrium. Interestingly, a link between location of the disease, symptoms, and patients’ age has been reported by Kobayashi [63], showing how external adenomyosis can be found more frequently in women of younger age affected by endometriosis, in comparison with internal adenomyosis.

Moreover, a new classification system for adenomyosis based on ultrasound findings has been proposed [43,64]; however, it has not yet achieved unanimous agreement.

#### 3.5.3. Sonohysterography

In adenomyosis, the infusion of saline into the endometrial cavity during sonohysterography can reveal continuity between the subendometrial cystic spaces, typical of this pathology, and the endometrial cavity. However, this technique cannot be considered a complete and exhaustive diagnostic method for adenomyosis [43,65].

#### 3.5.4. Hysteroscopy

Hysteroscopy allows for the identification of several endometrial signs suggestive of adenomyosis, such as endometrial hypervascularisation, strawberry pattern, endometrial defects, and subendometrial cysts [66,67,68]. However, this technique does not provide a definite diagnosis, but rather identifies certain suspicious signs, so further diagnostic investigation is always recommended for these patients [69].

#### 3.5.5. Elastography

The principle behind elastography involves slight compression of the tissue, producing displacement within the tissue. The stiffness of tissues is visualized with a gradation of colors ranging from red (the components with the greatest deformation, soft) to green (medium deformation) to blue (no deformation, harder). Elastography, when applied to TVUS, has been used to differentiate fibroids from adenomyosis.

Some authors have shown that adenomyotic tissues have lower stiffness than fibroids on elastography, with results consistent with MRI findings [70,71]. However, further studies are needed before this method can be validated as a diagnostic technique.

In conclusion, nowadays, noninvasive methods, such as MRI and transvaginal ultrasound, allow for the accurate diagnosis of adenomyosis without surgery. However, there are still many controversies regarding diagnostic criteria.

### 3.6. Differential Diagnosis

Differential diagnosis of adenomyosis in adolescence can be particularly challenging due to the nonspecific nature of symptoms and their overlap with other gynecological conditions that are more common in this age group. Understanding the shared symptomatology, the relative likelihoods of various conditions, and specific risk factors can guide clinicians in making an accurate diagnosis [72].

Adenomyosis, endometriosis, and primary dysmenorrhea frequently present with dysmenorrhea, chronic pelvic pain, and heavy menstrual bleeding (HMB) [73]. This overlap can complicate the diagnostic process. In adolescents, endometriosis is a leading cause of secondary dysmenorrhea and chronic pelvic pain, often presenting with pain that worsens over time and pain during intercourse (dyspareunia). In contrast, primary dysmenorrhea, affecting up to 90% of adolescent girls, is the most common cause of menstrual pain in this age group and may be more responsive to nonsteroidal anti-inflammatory drugs (NSAIDs). Although less common in adolescents than in older women, adenomyosis should still be considered, especially in cases of severe dysmenorrhea and abnormal uterine bleeding (AUB) that do not respond to standard treatments [29].

Uterine fibroids (leiomyomas) are an important differential diagnosis, though they are less common in adolescents than in adults. Nonetheless, they should be considered, especially with a family history or if imaging shows mass-like structures. Fibroids, which affect about 70–80% of women by age 50, are benign smooth muscle tumors that can cause symptoms similar to adenomyosis, such as heavy menstrual bleeding (HMB) and pelvic pain. It is crucial to differentiate fibroids, which are encapsulated and have typical pericapsular vascularization, from adenomyomas, which lack a capsule and show more pronounced intralesional vascularization [74,75,76,77].

Malignant myometrial pathologies, such as uterine sarcomas, are more prevalent in older women but can rarely present in younger patients with rapidly enlarging masses and atypical uterine bleeding. Differentiating these from benign conditions like adenomyosis requires careful imaging and, in some cases, histopathological evaluation [37,78].

Risk factors can significantly aid in distinguishing between these conditions. For instance, a family history of endometriosis or fibroids increases the likelihood of these conditions. Adolescents with early menarche, shorter menstrual cycles, or a history of HMB are at higher risk for endometriosis. Adenomyosis may be more likely in adolescents with a history of uterine surgeries, such as dilation and curettage (D&C), though this is less common in this age group. Polycystic ovary syndrome (PCOS), with a prevalence of 5–10% among women of reproductive age, can also present with menorrhagia and irregular menstrual cycles, but it is typically accompanied by hyperandrogenism and metabolic abnormalities, differentiating it from adenomyosis or endometriosis. Using imaging modalities to accurately distinguish adenomyosis from other conditions in adolescents is crucial for ensuring appropriate management and avoiding unnecessary interventions, which can improve quality of life and preserve future fertility.

### 3.7. Adenomyosis and Concomitant Endometriosis

The prevalence of endometriosis increases with age, but its true prevalence in adolescents remains unknown. It often goes undetected in about 65% of adolescent girls for several reasons [9,79,80]. In this age group, endometriosis typically presents as early-stage disease with superficial invasion and small endometriotic foci, which can be difficult to detect with imaging. Furthermore, transvaginal or transrectal ultrasound may not be feasible, as many teenagers are not sexually active [27,31].

Endometriosis should be suspected in cases of extreme use of nonsteroidal anti-inflammatory drugs (NSAIDs), a family history of the condition, frequent absenteeism from school during menstruation, or the early prescription of estrogen–progestin contraceptives during adolescence [80]. Endometriosis and adenomyosis often coexist in the same patient. Concurrent endometriosis is estimated to occur in about 3–10% of cases of adenomyosis. This highlights the importance of referring patients to specialized centers to reduce diagnostic delays and address potential fertility issues [81,82,83,84]. Zannoni et al. found a correlation between adenomyosis and endometriosis, particularly ovarian endometriosis. Moreover, the primary symptoms of endometriosis—dysmenorrhea, heavy menstrual bleeding, and dyspareunia—are similar to those of adenomyosis [85]. Millischer et al. found that among 121 patients with infiltrating endometriosis and/or ovarian endometriomas (OMAs) on MRI and severe dysmenorrhea, 17.3% also had adenomyosis [30]. Two studies reported that ultrasound signs of pelvic endometriosis were detected in 13% of the adolescent population [27,86]. These signs predominantly include ovarian endometriosis, but also adenomyosis and less frequently deep-infiltrating endometriosis (DIE), particularly of the uterosacral ligaments, similar to findings in the adult population [27]. Bourdon et al. observed two different profiles of adenomyosis: external and internal. External adenomyosis is more common in younger, nulligravid women and is associated with endometriosis, especially in those with a history of endometriosis surgery [87].

In conclusion, considering the hypothesis of the natural history of endometriosis, which may initially manifest with symptoms of pelvic adhesions and later evolve into adenomyosis, ovarian endometriomas, or deep infiltrating endometriosis, it is necessary to search for signs of adenomyosis in patients with endometriosis, and vice versa [85,88].

### 3.8. Adenomyosis and Autoimmune Diseases

Recent studies have explored the potential link between adenomyosis and autoimmune diseases, revealing a significant association with conditions as autoimmune thyroiditis, rheumatoid arthritis, and systemic lupus erythematosus (SLE). Toth et al. [89] found that patients with adenomyosis had a higher prevalence of autoimmune thyroiditis, with incidence rates ranging from 10% to 15%, compared to the general population’s 3–8% prevalence. This correlation suggests that the chronic inflammation characteristic of adenomyosis might play a role in the development of autoimmune disorders. Similarly, rheumatoid arthritis and SLE, both marked by systemic inflammation and immune dysregulation, have been observed more frequently in adenomyosis patients. Dimitriadis et al. [90] reported that approximately 20% of adenomyosis patients also suffer from rheumatoid arthritis, a notably higher percentage than in the general female population. The pathophysiological mechanisms underlying this association involve chronic inflammation leading to immune dysregulation, potentially triggering or worsening autoimmune diseases. Specifically, the ectopic endometrial tissue within the myometrium may act as an antigenic stimulus, provoking an abnormal immune response, including the production of autoantibodies and the activation of immune cells typically involved in autoimmune processes. Elevated levels of pro-inflammatory cytokines such as IL-6, TNF-α, and IL-1β are frequently observed in adenomyosis, creating an environment conducive to autoimmunity [91,92]. Furthermore, alterations in immune-modulating proteins like HLA-G, which are involved in immune tolerance, have been noted in adenomyosis, potentially impairing the immune system’s ability to differentiate self from non-self and facilitating the coexistence of adenomyosis with autoimmune conditions. Regarding adolescents, data are still limited; while adenomyosis is less common in this age group, when present, it may coincide with the emergence of autoimmune conditions, possibly due to shared immunological pathways or genetic susceptibilities that manifest during adolescence. This is particularly noteworthy given that the typical onset of both adenomyosis and autoimmune disorders generally occurs after adolescence. Hormonal imbalances, particularly elevated estrogen levels often found in adenomyosis, also play a critical role in modulating immune responses and may influence the severity and progression of autoimmune diseases. Furthermore, the association between inflammatory bowel disease (IBD) and adenomyosis represents an emerging area of interest in gynecology and gastroenterology. Though this link is less researched compared to other gynecological diseases like endometriosis, some studies suggest a potential correlation due to the shared inflammatory nature of both disorders [93,94,95]. Neri et al. found that the frequency of deep infiltrating endometriosis (DIE) and posterior adenomyosis is higher in IBD patients than in the general population. Similarly, Halis et al. [96] highlighted the overlap between gastrointestinal and gynecological conditions, suggesting that hormonal imbalances, chronic inflammation, and immune dysregulation in IBD may predispose patients to adenomyosis, emphasizing the need for multidisciplinary management. Further research is needed to clarify the prevalence of these associations in adolescents and to determine whether early intervention in adenomyosis could mitigate the risk or severity of autoimmune disorders. Nonetheless, these findings highlight the importance of considering autoimmune diseases in the diagnostic process for young patients presenting with symptoms indicative of adenomyosis.

### 3.9. Management

The treatment of adenomyosis is controversial and depends on the age of the patient, the impact of symptoms on quality of life, and her reproductive desire. Currently available evidence in the literature focusing on the clinical management of adenomyosis in adolescence age is limited. Potentially, as happens in the adult population affected by the disease, the gynecologist has both medical and surgical possibilities for the treatment. In the adolescent population, primary goals of therapy are the relief of symptoms and preservation of future fertility [97,98]. Current knowledge has largely been extrapolated from the treatment of adults with adenomyosis or endometriosis, as it is hypothesized that there are similarities between the diseases. Since data suggest that ovulation suppression is important for endometriosis regression, some theorize that adenomyosis may respond similarly [13]. The best therapeutic strategy to achieve is still an object of debate. One possibility is to identify adolescents with severe symptoms and to suppress the ovulation cycle from the ultrasound diagnosis or the onset of pelvic pain symptoms until the desire of conception [84]. Initial therapy for adenomyosis is based on the central suppression of hormone production, typically achieved using continuous oral contraceptives (COCs) [98]. Roshanak Mansouri et al. demonstrated regression of adenomyosis in four adolescents treated with hormonal suppression [99]. GnRH agonists represent an alternative treatment choice. Unfortunately, the extended use of GnRH analogues in young girls must be carefully evaluated. Indeed, we know that adult bone mass is not achieved until around 20 years of age, and for this reason reducing serum estrogen levels during adolescence could limit the optimal peak bone mineral density, and could be regarded as a possible risk factor for postmenopausal osteoporosis [100,101,102].

The therapeutic role of surgery for adolescents with chronic pelvic pain is debated. Although surgical management of adenomyosis has long been debated [103,104], in the adolescent population surgery alone is not always effective, as it addresses only the lesions rather than the underlying disease predisposition, leading to common recurrence of both symptoms and lesions [105]. Surgery could be considered in the rare cases of refractory focal lesions, especially if these lesions spare the uterine cavity, allowing for the preservation of fertility. Therefore, surgery remains a treatment option for selected young patients with pelvic pain that is refractory to medical therapy or for those with contraindications to hormonal medications [84]. A video article by Megan S. Orlando et al. presents a case report of a 16-year-old patient with chronic pelvic pain and ultrasound evidence of a 2.4 cm adenomyoma treated by laparoscopic resection. This case demonstrates a safe and effective resection of adenomyomas and treatment of refractory chronic pelvic pain [105].

## 4. Conclusions

Our work, although it does not represent the first paper focusing on the effects of adenomyosis in adolescence, provides a comprehensive overview on the topic, highlighting the difficulties related to both the diagnosis and the management of the pathology.

In conclusion, adenomyosis is a disease with early onset and early diagnosis is essential to avoid or delay surgery, enable medical management of the most severe symptoms, and preserve future fertility. The combination of clinical symptoms and non-invasive instrumental investigations (US and/or MRI) in expert hands can guide to the diagnosis.

Finally, we must not forget that medical therapy is essential to treat the symptoms and probably to reduce the progression of the disease.

## Data Availability

Data availability is not applicable to this article as no new data were created or analyzed in this study.
